# Neural Correlates of Verbal Episodic Memory and Lexical Retrieval in Logopenic Variant Primary Progressive Aphasia

**DOI:** 10.3389/fnins.2017.00330

**Published:** 2017-06-14

**Authors:** Khaing T. Win, John Pluta, Paul Yushkevich, David J. Irwin, Corey T. McMillan, Katya Rascovsky, David Wolk, Murray Grossman

**Affiliations:** ^1^Neuroscience Graduate Group, University of PennsylvaniaPhiladelphia, PA, United States; ^2^Neurology, Penn Frontotemporal Degeneration Center, Perelman School of Medicine, University of PennsylvaniaPhiladelphia, PA, United States; ^3^Radiology, Penn Imaging and Computing Science Lab, University of PennsylvaniaPhiladelphia, PA, United States; ^4^Neurology, Penn Memory Center, University of PennsylvaniaPhiladelphia, PA, United States

**Keywords:** logopenic primary progressive aphasia, Alzheimer's disease, verbal episodic memory, lexical retrieval, hippocampal subfields

## Abstract

**Objective:** Logopenic variant primary progressive aphasia (lvPPA) is commonly associated with Alzheimer's disease (AD) pathology. But lvPPA patients display different cognitive and anatomical profile from the common clinical AD patients, whose verbal episodic memory is primarily affected. Reports of verbal episodic memory difficulty in lvPPA are inconsistent, and we hypothesized that their lexical retrieval impairment contributes to verbal episodic memory performance and is associated with left middle temporal gyrus atrophy.

**Methods:** We evaluated patients with lvPPA (*n* = 12) displaying prominent word-finding and repetition difficulties, and a demographically-matched cohort of clinical Alzheimer's disease (AD, *n* = 26), and healthy seniors (*n* = 16). We assessed lexical retrieval with confrontation naming and verbal episodic memory with delayed free recall. Whole-brain regressions related naming and delayed free recall to gray matter atrophy. Medial temporal lobe (MTL) subfields were examined using high in-plane resolution imaging.

**Results:** lvPPA patients had naming and delayed free recall impairments, but intact recognition memory. In lvPPA, delayed free recall was related to naming; both were associated with left middle temporal gyrus atrophy but not MTL atrophy. Despite cerebrospinal fluid evidence consistent with AD pathology, examination of MTL subfields revealed no atrophy in lvPPA. While AD patients displayed impaired delayed free recall, this deficit did not correlate with naming. Regression analyses related delayed free recall deficits in clinical AD patients to MTL subfield atrophy, and naming to left middle temporal gyrus atrophy.

**Conclusion:** Unlike amnestic AD patients, MTL subfields were not affected in lvPPA patients. Verbal episodic memory deficit observed in lvPPA was unlikely to be due to a hippocampal-mediated mechanism but appeared to be due to poor lexical retrieval. Relative sparing of MTL volume and intact recognition memory are consistent with previous reports of hippocampal-sparing variant cases of AD pathology, where neurofibrillary tangles are disproportionately distributed in cortical areas with relative sparing of the hippocampus. This suggests that AD neuropathology in lvPPA may originate in neuronal networks outside of the MTL, which deviates from the typical Braak staging pattern of spreading pathology in clinical AD.

## Introduction

Logopenic variant primary progressive aphasia (lvPPA) is a neurodegenerative condition that is a form of primary progressive aphasia (PPA) characterized by core deficits in repetition and lexical retrieval, a process of linking the semantic representations of objects, actions, thoughts, and the like to their corresponding phonological word forms (Gorno-Tempini et al., 2011; Leyton and Hodges, 2013; Mesulam et al., 2013). According to the published diagnostic criteria (Gorno-Tempini et al., 2011), verbal episodic memory should be spared. However, evaluations of verbal episodic memory have been inconsistent in lvPPA (Rohrer et al., 2013; Flanagan et al., 2014). This is frequently assessed with delayed free recall, where participants name a list of recalled words after a delay period. However, recall may be confounded by the lexical retrieval deficit in lvPPA. Thus, their failure to retrieve the respective phonological word form of the target word can easily be confused with their inability to recall this target word on the memory paradigm. One group has assessed both verbal and non-verbal episodic memory in PPA patients, and shown verbal retrieval failures compared to relatively successful visual memory (Weintraub et al., 2013). However, this group studied a mixture of PPA variants (including agrammatic, logopenic, and semantic). Moreover, this study did not clarify whether delayed verbal free recall deficits in lvPPA specifically are due to hippocampal-mediated episodic memory difficulties or limited lexical retrieval.

Reports of hippocampal atrophy in lvPPA also have been inconsistent (Gorno-Tempini et al., 2011; Josephs et al., 2013; Mesulam, 2013), despite a statistical association of lvPPA with Alzheimer's disease (AD) pathology (Grossman, 2010). Likewise, the distribution of pathology in lvPPA has been unclear (Hu et al., 2010). Some have reported no observable difference in neurofibrillary tangle (NFT) density in hippocampus between lvPPA and clinical AD (Josephs et al., 2013), while others have reported minimal NFT pathology in the hippocampus of lvPPA (Gefen et al., 2012). These inconsistent reports on lvPPA cases may be due in part to the clinical heterogeneity of lvPPA. Recent findings (Mesulam et al., 2014a; Sajjadi et al., 2014; Leyton et al., 2015) showed that not all lvPPA cases are uniform: some displayed only lexical retrieval deficit with predominant atrophy in posterior-inferior temporal-parietal areas, while others displayed additional repetition deficit with prominent atrophy in left superior temporal gyrus. The third subgroup of lvPPA displayed mild deficits in single word comprehension with atrophy extending to the medial aspect of temporal cortex (Leyton et al., 2015).

No group has evaluated the role of lexical retrieval in verbal episodic memory in lvPPA, nor evaluated the anatomical basis for episodic memory difficulty in lvPPA. Here, we examined more closely the role of a lexical retrieval deficit in verbal episodic memory performance of lvPPA, and gray matter (GM) atrophy associated with these deficits in left lateral and medial temporal lobe (MTL) including hippocampus. Studies of neurodegenerative patients and fMRI studies of healthy adults have associated lexical retrieval with left middle temporal gyrus (Grossman et al., 2004; DeLeon et al., 2007; Baldo et al., 2013). Since both lvPPA (Gorno-Tempini et al., 2011; Leyton et al., 2015) and clinical AD patients (Grossman et al., 2004; Pekkala et al., 2013) have lexical retrieval difficulty, we predicted that left middle temporal gyrus would be associated with lexical retrieval in both lvPPA and clinical AD. We also hypothesized that, if a lexical retrieval deficit interferes with delayed free recall in lvPPA, left middle temporal gyrus atrophy would be associated with delayed free recall performance. By comparison, we predicted that delayed free recall performance in clinical AD would be associated with hippocampal atrophy. Because the hippocampus is comprised of different subfields, it is possible that subfields within the hippocampus in lvPPA are differentially affected. Traditional T1 imaging cannot detect subtle changes in hippocampal subfields (Yushkevich et al., 2014). To overcome this issue, we used high in-plane resolution T2 imaging to assess hippocampal subfields. This fine-grained analysis allowed us to examine whether delayed free recall in lvPPA and clinical AD could be due in part to selective atrophy of hippocampal subfields.

## Methods

### Participants

We studied 38 right-handed native English-speakers with lvPPA (*n* = 12) or clinical AD (*n* = 26), and 16 healthy controls [Mini-Mental State Examination (MMSE; Folstein et al., 1975) >27] with comparable gender (*X*^2^ = 0.08; *p* > 0.05), age [*F*_(2, 51)_ = 1.24; *p* > 0.05], and education [*F*_(2, 51)_= 0.49; *p* > 0.05] recruited from the Frontotemporal Degeneration Center at the University of Pennsylvania. lvPPA and AD patients were matched in MMSE (*U* = 94.5, *Z* = −1.95, *p* > 0.05) and disease duration (*U* = 153.50, *Z* = −0.08, *p* > 0.05). Because we were interested in whether lexical retrieval modulates verbal episodic memory, Boston Naming Test (BNT; Williams et al., 1989) performance in lvPPA and AD was matched (*U* = 111.00, *Z* = −1.43, *p* > 0.05). Diagnoses were established using published criteria for lvPPA (Gorno-Tempini et al., 2011) and clinical AD (McKhann et al., 2011) by board-certified neurologists (DJI, MG) based on a mental status examination. Clinically, lvPPA patients displayed word-finding and repetition problems while clinical AD patients displayed episodic memory difficulties. Exclusionary criteria included vascular disease, structural brain abnormalities such as hydrocephalus, medical diseases interfering with cognition, and primary psychiatric disorders. Nine of 12 lvPPA patients had cerebrospinal fluid (CSF) data: 8 with CSF ABeta42 <192 pg/ml [mean (S.D.) = 127.38 (27.43)] that is consistent with likely AD pathology and 1 without this criterion (ABeta42 > 192 pg/ml: 357 pg/ml). Fourteen of 26 clinical AD patients had available CSF data with ABeta42 <192 pg/ml [mean (S.D.) = 124.14 (20.67)].

### Behavioral methods

Neuropsychological testing included a 30-item BNT (Williams et al., 1989) to assess lexical retrieval; the delayed free recall component of the Philadelphia Verbal Learning Test (PVLT; Libon et al., 2011), a 9-word list-learning task (drawn from 3 semantic categories {tools, fruits, furniture}) with 5 learning trials, immediate recall and delayed recall probes, and recognition with equal numbers of foil types (semantic, interference, unrelated) to assess verbal episodic memory; and forward digit span (FDS; Wechsler, 1997) to assess repetition. To minimize the lexical retrieval component of verbal episodic memory, we also assessed the recognition component of PVLT (Libon et al., 2011) using d-prime, the difference between the z-transforms of hit rate and false alarm rate, to account for the false positive alarm rate. Shapiro-Wilk test showed that demographic variables were normally distributed (*p* > 0.05) but neuropsychological variables were not (*p* < 0.05). Hence, neuropsychological variables were assessed using non-parametric tests including *X*^2^, Mann-Whitney U, and Kruskal-Wallis.

#### Standard protocol approvals, registrations, and patient consents

All subjects completed a written informed consent procedure in accordance with the Declaration of Helsinki and approved by the institutional review board (IRB) of the University of Pennsylvania. The study was approved by the University of Pennsylvania's IRB.

### Imaging

We included subsets of lvPPA (*n* = 10; 8 with AD-CSF and 1 without) and AD (*n* = 26; 14 with AD-CSF and 1 without) patients who had available high-resolution T1 MRI scans, and an independent group of 17 healthy matched controls (gender: *X*^2^= 1.27, *p* > 0.05; age: *X*^2^= 0.38, *p* > 0.05; education: *X*^2^= 1.00; *p* > 0.05). These participants underwent a structural T1-weighted, 3-dimensional, spoiled gradient-echo sequence. Reasons for exclusion included health and safety (e.g., metallic implants, shrapnel, claustrophobia), intercurrent illness, scheduling, and transportation difficulty. Imaging was acquired within 6 months (μ = 2.6 months, σ = 2.6) of behavioral data. We used Advanced Normalization Tools (ANTs), a state-of-the-art pipeline, for all image processing, as described elsewhere (McMillan and Wolk, 2016).

#### T1 whole-brain imaging

Non-parametric permutation-based imaging analyses were performed with threshold-free cluster enhancement (TFCE) (Smith and Nichols, 2009) using the randomize tool in FSL (http://fsl.fmrib.ox.ac.uk/fsl/fslwiki). GM density was compared in lvPPA relative to controls. A *t*-test analysis was run with 10,000 permutations that is equivalent to a contrast corrected for multiple comparisons. The analyses were restricted to voxels containing GM using an explicit mask generated from the average GM probability map of all groups. We report clusters that survived a threshold of *p* < 0.005 (uncorrected with TFCE), and contained a minimum of 50 adjacent voxels.

To relate behavioral performance to significant GM atrophy, we used the randomize tool of FSL to compute regression analyses between lvPPA patients' performance on a target task and GM density in regions of the brain showing GM atrophy relative to controls. Permutations were run exhaustively up to a maximum of 10,000 for each analysis. We reported clusters surviving a height threshold of *p* < 0.05 uncorrected with TFCE and a minimum of 10 adjacent voxels. We used a very liberal statistical threshold purposefully to see if there is any possibility of a regression between episodic memory functioning and GM density in MTL of lvPPA.

#### Peak voxel region of interest (ROI)

Because we were interested to assess whether the region of left middle temporal gyrus identified in lvPPA was also implicated in AD performance, we extracted the statistically-significant peak voxel from the left middle temporal gyrus cluster identified in lvPPA (see below) and used this peak voxel as our label for left middle temporal gyrus in AD. GM density at this peak voxel in left middle temporal gyrus was calculated in AD patients and controls, and Spearman correlation assessed an association between GM density of left middle temporal gyrus and BNT and delayed free recall in AD.

#### T2 medial temporal lobe imaging

To determine whether lvPPA patients had subtle hippocampal atrophy that could have impacted their verbal episodic memory, we examined the subset of patients who had high-resolution T2 MRI scans (7 lvPPA {6 with AD-CSF, 1 without}, 19 AD (11 with AD-CSF, 1 without), and 17 demographically-matched controls. Images of each subject were labeled using the Automatic Segmentation of Hippocampus Subfields (ASHS) software (Yushkevich et al., 2014). This method utilizes a training and a segmentation pipeline that combines a multi-atlas label fusion (Wang et al., 2012) and a learning-based error correction module to produce a fully automated segmentation of Cornu Ammonis (CA), dentate gyrus (DG), and subiculum subfields along the entire length of the hippocampal formation, as well as segmentation of extrahippocampal structures, entorhinal (ERC), and perirhinal cortices (BA35 and BA36). Briefly, candidate segmentations of a subject's T2-MRI were obtained using high-dimensional mapping to multiple manually labeled atlas images, and then fused into a consensus segmentation, taking into account the similarity between a subject's image and atlas images. Patterns of systematic segmentation errors are learned *a priori* using training data, and are corrected in a further post-processing step, to generate the final segmentation. Reliability of automated labeling for these subfields is generally high, as reported (Yushkevich et al., 2014), but CA2/3 were excluded since their segmentation was not reliable. Volumetric measures of the hippocampal subfields and thickness of extrahippocampal subfields were extracted for quantitative comparisons across cohorts. Thickness was used for extrahippocampal subfields since these subfields were not segmented throughout the entire anterior-posterior axis of MTL and we needed to normalize the volume by the number of segmented slices. Spearman correlation analyses were performed to relate MTL subfield atrophy to BNT as well as delayed free recall.

## Results

### Behavioral analysis

Compared to controls (Table 1), worse MMSE was observed in lvPPA (*U* = 18.00, *Z* = −3.83, *p* < 0.001) and AD (*U* = 2.00, *Z* = -5.42, *p* < 0.001). However, lvPPA and AD patients were matched in MMSE (Folstein et al., 1975) (*U* = 94.5, *Z* = −1.95, *p* > 0.05) and disease duration (*U* = 153.50, *Z* = −0.08, *p* > 0.05). Consistent with their clinical phenotype, lvPPA patients were more impaired on BNT (*U* = 47.00, *Z* = −2.31, *p* < 0.05) and FDS (*U* = 21.50, *Z* = −3.54, *p* < 0.001) than controls. FDS also was worse in lvPPA than AD (*U* = 73.00, *Z* = −2.66, *p* < 0.05). Though recognition memory was intact in lvPPA relative to controls (*U* = 66.50, *Z* = −1.49, *p* > 0.05), these patients exhibited worse delayed free recall relative to controls (*U* = 39.00, *Z* = −2.69, *p* < 0.01). A correlation analysis revealed that BNT was associated with delayed free recall in lvPPA (*rs* = 0.721, *p* = 0.019) but BNT was not associated with recognition memory (*rs* = 0.302, *p* = 0.34). Intact verbal recognition memory suggested relatively preserved verbal episodic memory in lvPPA, and a common cognitive mechanism underlying impairments in lexical retrieval and delayed free recall in lvPPA.

**Table 1:** Mean (standard deviation) demographic and clinical features of the cohorts.

**Measure**	**lvPPA (*n* = 12)**	**AD (*n* = 26)**	**Healthy seniors (*n* = 16)**
**DEMOGRAPHIC FEATURES OF THE COHORTS**			
Gender (Male/Female)	5/7	14/12	7/9
Age, years	63.10 (8.80)	64.73 (7.78)	69.00 (9.13)
Education, years	16.08 (4.12)	15.19 (2.68)	15.94 (2.49)
Disease duration, years	3.75 (2.38)	3.73 (2.00)	N/A
**NEUROPSYCHOLOGICAL PERFORMANCE**			
Mini mental state exam (max = 30)	25.67 (3.20)	23.69 (2.19)	29.63 (0.72)
Forward digit span (# digits)	4.67 (1.07)	5.88 (1.45)	6.68 (1.20)
Boston naming test (max = 30)	23.75 (6.77)	22.85 (5.11)	28.19 (1.97)
Recognition memory (d-prime)	2.77 (0.57)	1.88 (1.04)	3.04 (0.24)
Delayed free recall (max = 9)	4.58 (3.15)	1.73 (2.51)	7.69 (1.30)

Relative to controls, AD performed worse on BNT (*U* = 52.50, *Z* = −4.05, *p* < 0.001), delayed free recall (*U* = 22.00, *Z* = −4.91, *p* < 0.001) and recognition memory (*U* = 78.50, *Z* = −3.39, *p* < 0.005) but had intact FDS (*U* = 133.00, *Z* = −1.68, *p* > 0.05). Relative to lvPPA, AD had worse delayed free recall (*U* = 70.50, *Z* = −2.76, *p* < 0.01) and recognition memory (*U* = 89.5, *Z* = −2.10, *p* < 0.05). Unlike lvPPA, there was no association between delayed free recall and BNT in AD (*rs* = 0.110, *p* = 0.591).

### Imaging analysis

#### Whole-brain imaging

**Figure 1:**
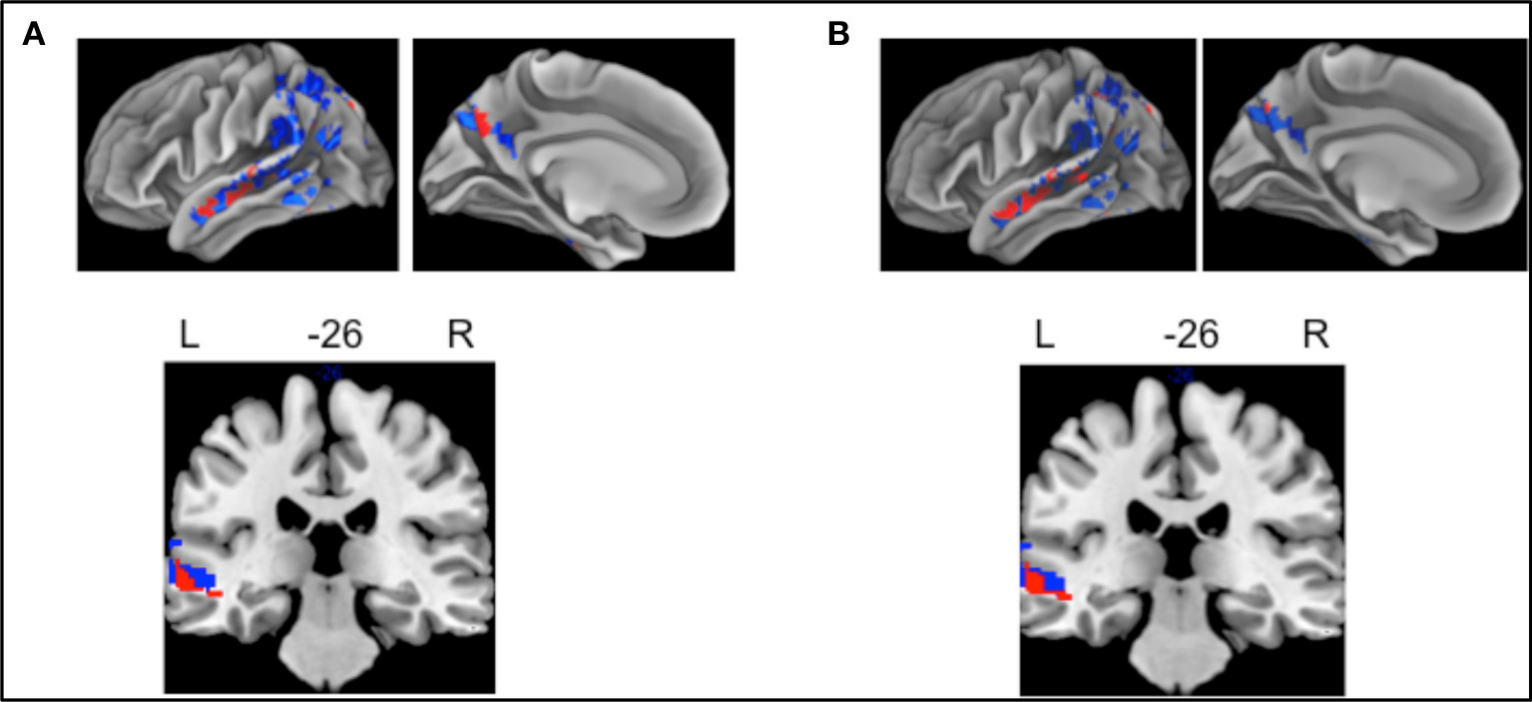
Whole Brain Atrophy and Regression in lvPPA. Pattern of atrophy of gray matter (GM), shown in blue (surface rendering and coronal slice), in lvPPA compared to controls. Atrophy was found only in the left hemisphere; these areas include middle temporal and parietal areas, significant at *p* < 0.005 (uncorrected with threshold-free cluster enhancement). **(A)** Decreased performance on BNT related to GM atrophy is shown in red. **(B)** Decreased performance on delayed free recall related to GM is shown in red. Left middle temporal gyrus regression for both BNT and delayed free recall was shown in the coronal slice.

We examined whether there was a shared neuroanatomic substrate for BNT and delayed free recall in lvPPA. Compared to controls, lvPPA displayed GM atrophy only in left middle temporal gyrus (Figure 1), while the hippocampus was spared. Regression was used to relate behavioral performance to GM atrophy in lvPPA; decreased performance on both BNT (Figure 1A) and delayed free recall (Figure 1B) was related to left middle temporal gyrus atrophy.

#### Region of interest analysis

GM density at the peak voxel of left middle temporal gyrus cluster associated with BNT and delayed free recall in the whole-brain regression analysis described above was extracted for every participant of the cohort. GM density of this peak voxel was reduced in lvPPA compared to controls (*U* = 61.00, *Z* = −2.01, *p* < 0.05) and in AD compared to controls (*U* = 114.00, *Z* = −2.52, *p* < 0.05). Reduced GM density at this left middle temporal gyrus voxel in AD was associated with decreased performance on BNT (*r* = 0.537, *p* = 0.006) but not with delayed free recall (*p* > 0.05).

#### MTL subfield imaging

**Figure 2:**
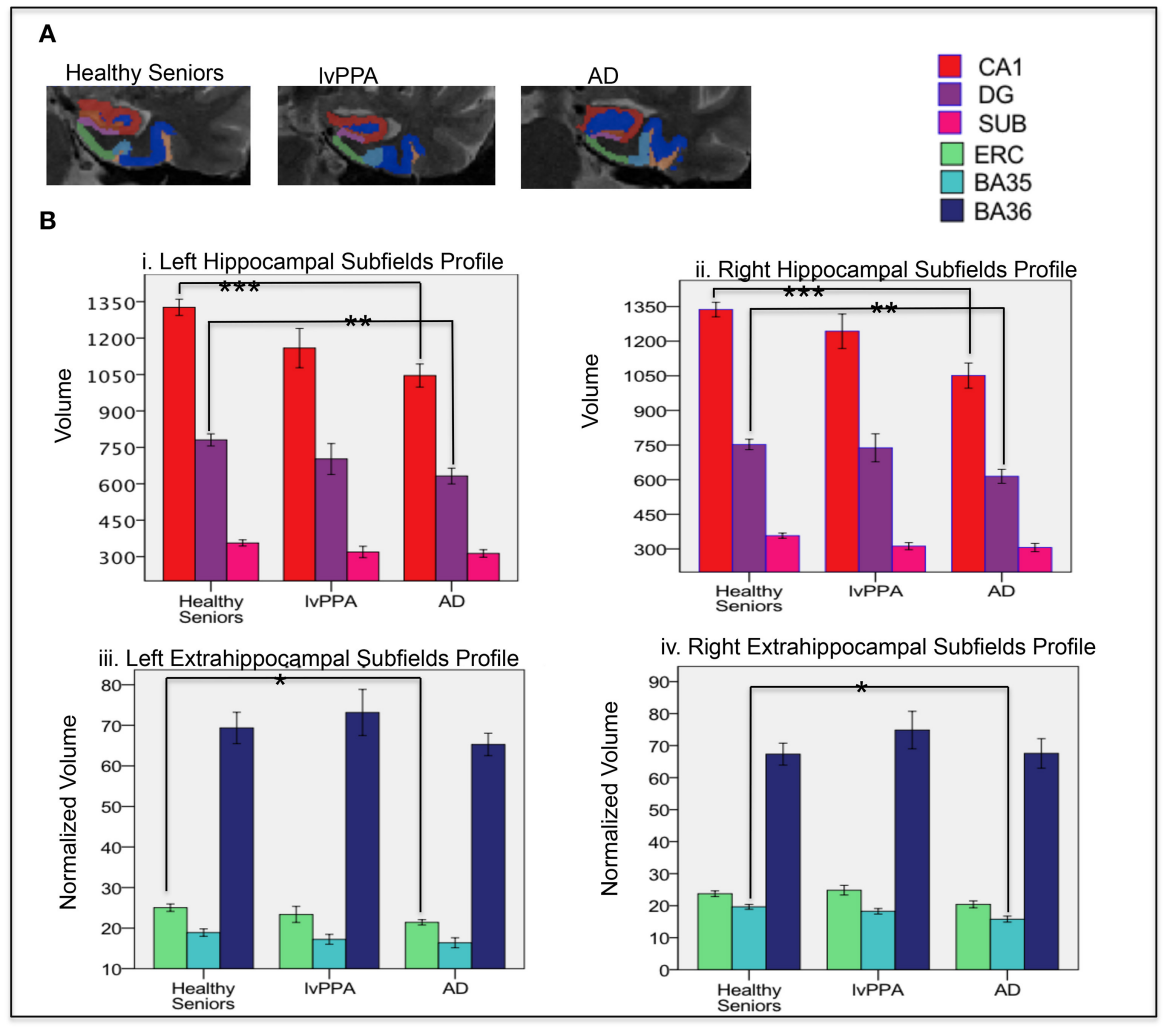
**(A)** A representative segmentation of left MTL subfields in one of the participants in each group (healthy senior, lvPPA, AD) shown in a coronal view. **(B)** Profile of volume of left and right hippocampal and extrahippocampal subfields across cohorts. ^*^p < 0.05, ^**^p < 0.005, ^***^p < 0.001. CA, Cornu Ammonis; DG, dentate gyrus; SUB, subiculum; ERC, entorhinal. and perirhinal cortices (BA35 and BA36).

We examined whether hippocampal (CA1, DG, and subiculum) and extra-hippocampal subfields (ERC, BA35, and BA36) were differentially affected in lvPPA (Figure 2A). Consistent with whole-brain regression results, there was no significant difference in hippocampal and extrahippocampal subfields between lvPPA and controls. However, AD patients had differential MTL subfield atrophy compared to controls. This included bilateral CA1 and DG [Figure 2B; left CA1 (*p* < 0.001), right CA1 (*p* < 0.001), left DG (*p* < 0.005), right DG (*p* < 0.005). AD also showed atrophy in extra-hippocampal subfields (Figure 2B), including left ERC (*p* < 0.05) and right BA35 (*p* < 0.01)].

Spearman correlation was used to assess an association between MTL subfields and behavioral performance. Neither BNT nor delayed free recall was associated with MTL subfields in lvPPA. In AD, both left CA1 (*rs* = 0.543, *p* = 0.016) and right CA1 (*rs* = 0.473, *p* = 0.022) were associated with delayed free recall, but not BNT (*p* > 0.05).

## Discussion

The status of verbal episodic memory performance in lvPPA is unclear. In this study, we aimed to elucidate whether the core language deficit in lexical retrieval in lvPPA interferes with their verbal episodic memory functioning and whether hippocampus disease also contributes to episodic memory difficulty, given the statistical likelihood of underlying AD pathology (Josephs et al., 2008; Grossman, 2010; Mesulam et al., 2012). We found impaired lexical retrieval in lvPPA, and this correlated with their delayed verbal free recall performance. This was mediated by a common neural substrate in left middle temporal gyrus, but did not appear to be associated with MTL. Although CSF analyses indicated that likely AD pathology was present in a majority of lvPPA cases, detailed examination of hippocampal subfields failed to reveal any atrophy in lvPPA relative to controls. By comparison, clinical AD patients had atrophy in MTL subfields, which was associated with verbal episodic memory difficulty, while BNT was associated with left middle temporal gyrus. These findings are consistent with other reports of quantitative pathologic evidence of a hippocampal-sparing aphasic variant with AD pathology, which has disproportionate amount of NFT in cortical areas with relative sparing of the hippocampus (Janocko et al., 2012). The hippocampal-sparing variant of AD with primarily progressive aphasia due to prominent neocortical AD pathology accumulation is different from typical amnestic AD, where the NFT distribution follows the Braak staging pattern (Braak and Braak, 1991), of significant NFT burden in MTL structures, including the hippocampus, relative to NFT distribution in the cortex. These different clinical and pathological variants of AD call to question the hypothesis that pathology originates in the MTL of all patients with AD pathology.

According to the 2011 criteria for PPA (Gorno-Tempini et al., 2011), patients are diagnosed with lvPPA if they exhibit impaired lexical retrieval and repetition as their primary deficit, with relatively preserved episodic memory. Given that lvPPA is a form of aphasia, most studies have focused on the language domain and have not evaluated the role of memory performance in the difficulties associated with diagnosing lvPPA. Evaluating verbal episodic memory performance in lvPPA may pose a challenge. Indeed, reports of verbal episodic memory performance in lvPPA have been inconsistent, with some reporting impaired memory (Flanagan et al., 2014; Piguet et al., 2015; Ramanan et al., 2016) and one study observing equally impaired verbal episodic memory and recognition memory in AD and lvPPA patients who also had visual memory difficulty (Ramanan et al., 2016), while others found no deficit (Weintraub et al., 2013; Mesulam et al., 2014b).

Verbal episodic memory is typically tested using delayed free recall, which requires lexical retrieval and production, and therefore verbal episodic memory can be confounded by the lexical retrieval deficit observed in lvPPA. However, previous work has not indicated whether verbal episodic memory difficulty is associated with impaired lexical retrieval. We found verbal episodic memory difficulty in lvPPA and that this deficit was correlated with their impaired lexical retrieval, raising the possibility that lexical retrieval difficulty may underlie in part the verbal episodic memory deficit that is reported at times in lvPPA. We also found that our lvPPA cohort had preserved verbal recognition memory, and we hypothesize that this was because this form of verbal episodic memory testing does not require lexical retrieval and production. While it is possible that successful recognition memory performance was due in part to the fact that this is an easier task than delayed free recall, it is noteworthy that the clinical AD patients matched the lvPPA patients in MMSE, yet lvPPA patients were significantly less impaired in their recognition memory than AD. Another group reported similar findings, where PPA patients were impaired on verbal recall, but their performance on verbal recognition memory was near ceiling compared to controls (Weintraub et al., 2013). Additional work is needed to evaluate episodic memory performance in lvPPA.

Our findings associated lexical retrieval difficulty in lvPPA with atrophy of left middle temporal gyrus. This area has been widely implicated in lexical retrieval in lvPPA (Henry and Gorno-Tempini, 2010; Leyton and Hodges, 2013; Mesulam et al., 2013, 2014a) as well as AD (Harasty et al., 1999; Grossman et al., 2004; Apostolova et al., 2008) and stroke (DeLeon et al., 2007; Indefrey, 2011). Moreover, delayed free recall performance in lvPPA was related to the same area of left middle temporal gyrus, underlining the contribution of impaired lexical retrieval to the verbal episodic memory deficits of lvPPA. Moreover, there was no hippocampal atrophy in lvPPA that could have explained any memory difficulty. Our study also found that lexical retrieval difficulty in AD is associated with the same cluster of left middle temporal gyrus that was atrophied in lvPPA. Unlike lvPPA, delayed free recall performance in AD was not related to left middle temporal gyrus.

Despite the presence of likely AD pathology in most of our lvPPA cohort, detailed analysis reveal no observable changes in hippocampal and extrahippocampal subfields compared to controls, while AD patients showed hippocampal subfield atrophy that was associated with their verbal episodic memory deficit. Involvement of the hippocampus in the neuroimaging and pathological literature in lvPPA has been inconsistent. One report described greater NFT burden in language-related areas and throughout the left hemisphere than entorhinal cortices in lvPPA, and that NFT deposition is greater in the left peri-Sylvian language cortices than typical AD patients (Gefen et al., 2012). Others reported no observable difference in NFT density in the hippocampus in lvPPA compared to AD, consistent with their imaging result, although NFT density ratio in temporoparietal areas relative to hippocampus is higher in lvPPA (Josephs et al., 2013). MMSE of lvPPA reported in this paper was in the moderately impaired range (14.3 ± 7.7), reflecting patients with considerably more cognitive impairment than our cohort, and this may explain in part the discrepancy with findings of the present study where patients had milder cognitive impairment (MMSE: 25.67 ± 3.2). Other sources of discrepancy in the literature may be due in part to the relatively small sample sizes of lvPPA, different severity and disease duration of patient groups, and pathological observations typically obtained many years after the clinical phenotype has been ascertained. Nevertheless, paralleling our findings, a functional connectivity study demonstrated that the language network encompassing left posterior temporal areas is more affected in lvPPA than in AD, while the ventral default mode network associated with episodic memory was affected in AD more than lvPPA (Whitwell et al., 2015). Longitudinal imaging studies of lvPPA patients have suggested that the atrophy initially involving the left temporoparietal region subsequently spreads to include other left hemisphere regions (Rohrer et al., 2010; Rogalski et al., 2011), although the presence of likely AD pathology was assessed only in a small number of cases. Hippocampal-sparing variant of AD cases has been identified as one of the three AD subtypes [amnestic AD (75% out of 889 cases), hippocampal sparing (11%), and limbic predominant (14%)] in autopsy series of patients with AD pathology (Murray et al., 2011). Our findings, together with these suggestive studies, raise the possibility of a variant of AD where pathology does not originate in the MTL, and suggest that the lvPPA phenotype may be a marker of this variant. Findings such as these warrant more detailed examination of hypotheses concerned with spreading pathology in neurodegenerative diseases such as AD.

Some limitations must be considered when interpreting our data. First, atrophy in our lvPPA cohort was limited to left middle temporal gyrus. Other neuroimaging studies have shown a greater extent of atrophy encompassing left temporal-parietal junction (Gorno-Tempini et al., 2004; Migliaccio et al., 2009). When using a more liberal threshold (*p* < 0.01, uncorrected), we also observed a similar atrophy pattern in left temporal and parietal areas area consistent with other neuroimaging studies. However, we did not observe left MTL atrophy even at this more liberal threshold in our lvPPA cohort. Second, while our cohorts were carefully matched, our sample size was relatively small. Larger cohorts of lvPPA patients are needed to assess these brain-behavior relationships more reliably. Although our patients had a typical phenotype of lvPPA that is statistically associated with AD pathology, two of our cases did not have CSF available that could have provided biomarker evidence of AD. Additional longitudinal studies would be helpful in resolving discrepancies concerning disease severity across studies. Lastly, our lvPPA cohort was relatively young, as was our matched AD group. Caution thus must be exercised in generalizing our findings to late-onset patients with lvPPA and AD.

With these caveats in mind, we conclude that lexical retrieval difficulty in lvPPA interferes with verbal episodic memory functioning, and left middle temporal gyrus disease may be contributing to this common cognitive mechanism. No hippocampal atrophy was evident despite the presence of likely AD pathology in lvPPA. Verbal delayed free recall thus can be confounded by lexical retrieval difficulty in lvPPA, and this confound may be circumvented in part with recognition memory testing. Although both our AD and lvPPA cohorts were cognitively comparable, the cognitive and anatomical profile of lvPPA is distinct from AD, where the apparent memory deficit was mediated by hippocampal disease and was not modulated by lexical retrieval difficulty or left middle temporal gyrus atrophy. These distinctions suggest that lvPPA may be a marker for an atypical, hippocampal-sparing variant of AD pathology that may not originate in the MTL.

## Author contributions

KW: Study concept and design; acquisition of data; analysis and interpretation of data; drafting, editing and revising the manuscript. JP: Manual segmentation of MTL subfields for developing atlases for automatic segmentation algorithm. PY: Develop the automatic segmentation of medial temporal lobe subfield algorithm. DI: Patient recruitment; revision of manuscript. CM: Interpretation of data; revision of manuscript. KR: Consultation for neuropsychological measures. DW: Study concept and supervision; revision of manuscript. MG: Patient recruitment; study concept, supervision and funding; interpretation of data; revision of manuscript.

### Conflict of interest statement

The authors declare that the research was conducted in the absence of any commercial or financial relationships that could be construed as a potential conflict of interest.
